# Parent and Teacher Training Increases Medication Adherence for Primary School Children With Attention-Deficit/Hyperactivity Disorder

**DOI:** 10.3389/fped.2020.486353

**Published:** 2020-11-09

**Authors:** Xiaofei Zheng, Li Shen, Lian Jiang, Xiao Shen, Ying Xu, Guangjun Yu, Yu Wang

**Affiliations:** ^1^Department of Child Health Care, Shanghai Children's Hospital, Shanghai Jiaotong University, Shanghai, China; ^2^School Affiliated With Shanghai Caoyang No. 2 High School, Shanghai, China

**Keywords:** training, parent, teacher, ADHD, adherence, children

## Abstract

**Objective:** Attention-deficit/hyperactivity disorder (ADHD) is a common neurobiological disorder for which effective and safe medication is recommended as first-line treatment. However, many parents and teachers do not believe that ADHD is a disorder or do not accept medication treatment in China. Treatment is often short term or intermittent. Our study aimed to investigate the clinical effect of employing a 4-week, session-based training for both parents and teachers in improving medication adherence for primary school children with ADHD.

**Methods:** From January 2018 to December 2018, a total of 5,118 primary school children were screened. Among 211 children diagnosed with ADHD, 116 were assigned to the intervention group and 95 to the control group. This study provided systematic training for parents and teachers in the intervention group. The training consisted of education about the disorder and ADHD behavioral intervention for both parents and teachers as well as classroom management techniques for just the teachers. A cluster randomized controlled trial (RCT) was conducted to investigate the effect of this training at 6 months follow-up. The study determined medication adherence using a questionnaire and scoring with a rating scale at baseline and at the 6 month follow-up endpoint. The questionnaire was self-report.

**Results:** The study population had a relatively low rate of attention deficit hyperactivity disorder (4.1%) compared to the generally accepted prevalence. After the training, more parents and teachers believed that ADHD is a neurobiological disorder and that medication is the first line treatment. At 6 months follow-up, the Medication Adherence Report Scale (MARS) score for the intervention group was 22.8 ± 0.75 and 16.5 ± 1.63 for the control group (*t* = 5.217, *P* < 0.01). Based on parents' reports and medical records, 82 children (70.69%) were continuously taking medication for 6 months in the intervention group, while only 35 children (36.84%) were doing so in the control group. In the intervention group, the mean SNAP-IV score was 1.98 ± 0.42 at baseline but 0.99 ± 0.31 at 6-month follow-up. In the control group, the mean SNAP-IV score was 1.89 ± 0.47 at baseline but 1.37 ± 0.42 at 6-months follow-up (*F* = 2.67, *P* = 0.009). Factors influencing medication adherence for children with ADHD were parent's beliefs, teacher's beliefs, socioeconomic status, adverse effect, insurance coverage, gender, and trust of the medical system.

**Conclusions:** Our findings indicate that comprehensive training programs improve the understanding of ADHD and medication adherence for both children's parents and teachers, providing a promising approach for improving clinical efficacy for children with ADHD.

## Introduction

Attention-deficit/hyperactivity disorder (ADHD) is a common neurobiological disorder, characterized by symptoms of overactivity, impulsivity, and inattention (1). Approximately 7% of children are estimated to be affected by ADHD worldwide (2). Effective and safe medication is recommended as the first-line treatment for ADHD (3). Primary medications in China include methylphenidate (MPH) and atomoxetine (ATX). Stimulants such as MPH increase dopamine in the brain generally, while ATX leads to a selective increase in norepinephrine and an indirect increase of dopamine in the frontal cortex (4). Medication adherence is important because untreated ADHD can result in serious consequences with lifelong effects, such as school difficulties, fewer friends, arrests, unwanted pregnancy, and alcohol or drug abuse (5). It has been reported that medication treatment for children with ADHD can reduce the risk of criminality, comorbid psychiatric disorders, and substance abuse in adolescence and adulthood (6, 7). For example, high adherence to ADHD medication has been reported to result in higher academic achievement among children with ADHD (8, 9).

In recent years, with the expectation and hope that early treatment will diminish poor mental health and psychosocial outcomes in adolescence and adulthood, ADHD interventions have largely targeted children of primary school age (10). In China, the prevalence of ADHD among children and adolescents is 6.26% (11) or 23 million affected individuals. At present, the consultation rate of ADHD patients in China is low, at only 10%, and only about one third of families receive medication treatment (11). So far, the compliance of children with ADHD in China is relatively poor. A considerable number of children with ADHD have missed the ideal timing for treatment, leading to the occurrence or a significant increase of comorbidities before visiting doctors (11). In addition, increased age leads to increased ADHD-related comorbidities or secondary learning difficulties, along with oppositional defiant disorder. As a result, therapy for ADHD becomes more challenging.

However, many parents and teachers do not accept or continue medication treatment in China. They do not believe that ADHD is a neurobiological disorder or that medication is safe (11). For children, healthcare decisions are usually made by their parents, and beliefs and attitudes may differ widely. However, for other families, medication treatment is unacceptable. They prefer implementing behavioral strategies and other non-medication strategies (11). Choices about using medication are complex, with more recent work focusing on beliefs and attitudes that shape patient preferences (12, 13). In addition, effectiveness and adverse effects, two-parent families, higher socioeconomic status, insurance coverage, Caucasian racial background, and combined subtype are all important factors of increased adherence (14).

In addition to parental decisions, teachers are an important part of children's school environments. Whether children's ADHD can be detected earlier depends to some extent on teacher beliefs about ADHD. In the clinical evaluation stage, doctors need to refer to teachers' opinions. In the treatment phase, medication dosing should also be adjusted according to teachers' feedback on school performances (15). Training for parents and teachers of children with ADHD is necessary and urgent in China. However, to our knowledge, few studies exist that examine the effect of parent/teacher training for children with ADHD in China. Our study was undertaken within primary schools to systematically implement parent and teacher training targeted at increasing medication adherence for children with ADHD. A cluster randomized controlled trial (RCT) was conducted to investigate the effect of training for parents and teachers of children with ADHD at 6 months follow-up.

## Materials and Methods

This study was a cluster randomized controlled trial (RCT) to investigate the effect of parent and teacher training at 6 months follow-up. It consisted of three parts: ADHD screening and diagnosis, parent and teacher training, and evaluation of the effect of the training.

### Study Design and Population

A RCT was carried out at 10 primary schools (children aged 6–11 years) located in Putuo District, Shanghai, China. First, an invitation and information letter was sent to each school's in-house health care professional (iHCP) and the headmaster, informing them about the study. Hospital pediatricians conducted a meeting for both teachers and parents to introduce the purpose of this project and the type of intervention. Parents who wanted to participate were asked to complete and return the informed consent letter within 3 days.

**Figure 1:**
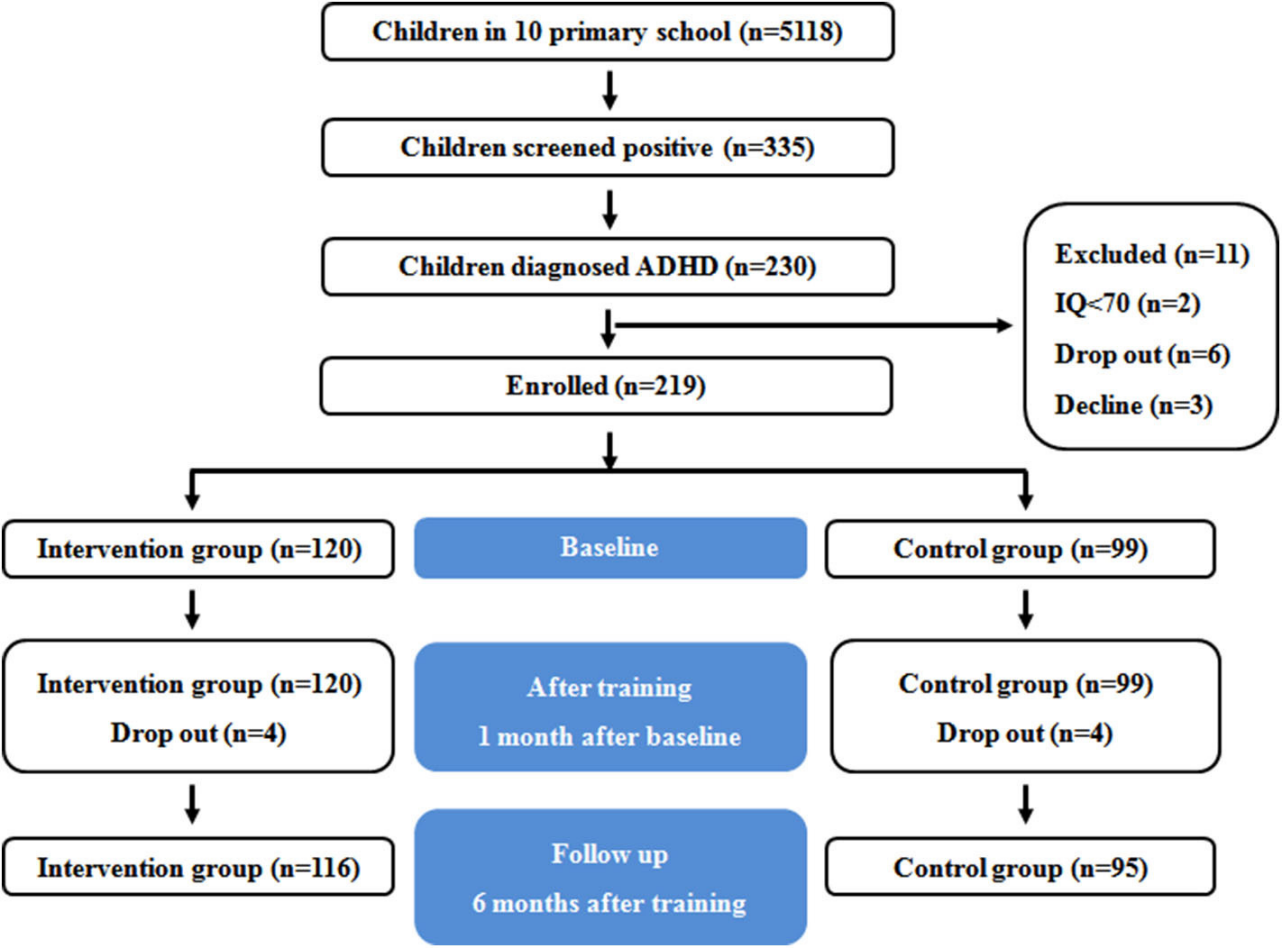
A flow diagram of participants.

Students were enrolled in this study between January 2018 and June 2018. Conner's Parent Symptom Questionnaire (PSQ) and Conner's Teacher Rating Scale (TRS) were used for ADHD screening. Children whose results on both scales were positive received a call or a notification to have a pediatric examination in Shanghai Children's Hospital. Within 1 month of issuing the screening questionnaire, pediatricians identified 6- to 11-year-old children diagnosed with ADHD who also met inclusion or exclusion criteria. Hospital pediatricians sent a program invitation and informed consent form to the parents of these children. There were 116 children diagnosed with ADHD in the intervention group and 95 in the control group. A flow diagram of participants is shown in Figure 1.

### Inclusion and Exclusion Criteria

Children were diagnosed with ADHD according to the Diagnostic and Statistical Manual of Mental Disorders, fifth edition (DSM-5) (16), IQ ≥70 on the Wechsler Intelligence Scale (WISC), aged 6- to 11-years old, and with no prior ADHD medication use. Additional inclusion criteria were that parents or primary caregivers accepted medication therapy (methylphenidate or atomoxetine), can read and write the Chinese language, were legally able to sign informed consent, and signed the informed consent. Children who met the above criteria were invited to take part in this study.

Exclusion criteria were allergy to methylphenidate or atomoxetine, comorbidity with autism spectrum disorder, schizophrenia, epilepsy, head injury, or verified neurological disorder, intellectual disability (IQ <70), sensory retardation (hearing/vision problems), and unwilling or unable to give consent. Both the intervention group and the control group were treated with medication. This study provided a multimodal treatment for patients and teachers in the intervention group.

### Randomization and Blinding

After the screening and diagnosis of students, the research team used a random number table method to divide the 10 primary schools participating in the study into the intervention group and the control group (5 schools in each group) stratified by school size. This occurred at the school, not at the class or individual level, in order to avoid contamination between individuals. Outcomes were obtained through questionnaires filled out by parents or teachers, and a limitation of the study is that teachers' and parents' assessment (questionnaires) are non-blinded. Statistical analysis was blinded to school intervention status when performing analysis of data results.

### Parent Training

Parent training involved 4 weekly 2-h sessions, delivered in primary schools and consisted of: (1) knowledge about ADHD: etiology, manifestation, harm, and treatments; (2) medication: side effects and how to deal with them, teaching parents how to evaluate their children's behavior with the SNAP-IV scale; (3) teaching parents behavioral strategies to manage conduct problems, typically apply principles of positive and negative reinforcement; and (4) teaching parents to combine procedures and behavior management techniques, which can constructively target impairments in underlying processes, such as attention, impulse control, self-regulation, and working memory (17).

### Teacher Training

Teacher training involved 4 weekly 2-h sessions, delivered in primary schools and conducted simultaneously with parent training. The teacher training was delivered in primary schools and conducted concurrently with parent training. It consisted of: (1) knowledge about ADHD: etiology, manifestation, harm, treatment, and follow-up; (2) behavioral strategies to manage conduct problems, typically applying principles of positive and negative reinforcement; (3) classroom behavior management; (4) teaching teachers how to use scaffolding to promote the development of self-regulation in children with ADHD, and how to take on the role of skilled tutor to create settings and occasions in a positive emotional climate, which can produce playful and reciprocal interactions (18).

When we designed training for parents and teachers, we referred to Family School Success (FSS) and simplified it. FSS is an integrative and novel psychosocial intervention for children with ADHD, which combines components of efficacious interventions to improve children's behavioral and academic functioning at home and school settings (19). FSS aims to improve parental involvement in education and parent–teacher collaboration in order to improve children's behavior and academic performance. However, there are currently only 135,524 pediatricians in China, just four per 10,000 children (20). Therefore, when we did the behavior management training, we simplified the course to make the training feasible and effective in China. Our goal is to extend the training program to more areas of China in order to validate our findings with a larger-scale population.

### Statistical Analysis

The Statistical Package for the Social Sciences (SPSS) 21.0 was used. Descriptive statistics, means, standard deviations and frequencies were calculated. *T*-test was used for continuous variables and chi-square tests for categorical variables between the intervention group and the control group. Multiple regression models were created in which the scores of the total MARS were used as dependent variables.

### Questionnaires

#### Conner's Parent Symptom Questionnaire and Conner's Teacher Symptom Questionnaire

##### Conner's Parent Symptom Questionnaire (PSQ)

PSQ is used to assess ADHD symptoms in primary school patients. It is a 48-item, parent-reported questionnaire using a four-point scale ranging from 0 to 3 and six subscales: conduct problems, learning problems, psychosomatic, hyperactivity–impulsivity, anxiety, and ADHD index (21). If the ADHD index is ≥1.5, it suggests that the patient may have ADHD.

##### Conner's Teacher Rating Scale (TRS)

TRS is a widely used instrument for evaluating behavioral problems in children and adolescents. It contains a 28-item teacher-reported questionnaire using a four-point scale ranging from 0 to 3 and four subscales: conduct, hyperactivity, inattention, and ADHD index (22). If the ADHD index is ≥1.5, it suggests that the patient may have ADHD.

#### Medication Adherence Report Scale

The Medication Adherence Report Scale (MARS) questionnaire is used to assess adherence to prescribed medication, which includes five statements using a five-point scale ranging from 1 to 5. One statement is related to the subscale of unintentional non-adherence (forgot to take them) and four statements are related to intentional non-adherence behaviors (altering the dosage, stopping medication use, missing a dose, taking less than instructed) (23). Higher scores indicate higher levels of medication adherence.

#### Swanson Nolan and Pelham, Version IV Scale Score

The Chinese Version of Swanson Nolan and Pelham, Version IV Scale (SNAP-IV) is used as the measure for the severity of ADHD symptoms. It consists of 26 items using a four-point scale ranging from 0 to 3 and three subscales: inattention, hyperactivity, and impulsivity (24). The primary outcome measure is the difference in the severity of ADHD core symptoms as measured by the SNAP-IV scales between the intervention group and the control group at baseline and 6 months follow-up.

## Results

### Baseline

From the 5,118 children who completed the screening questionnaire, 335 (6.55%) were positive from the screening. From those children, 230 (68.66%) were physician- diagnosed with ADHD. However, 19 students were excluded: 5 had intellectual disability, 1 dropped out of school, and 13 declined to participate in this study. One hundred sixteen children were assigned to the intervention group, and 95 were assigned to the control group.

Table 1 shows the demographics of children in both groups. There were no statistically significant baseline differences between the intervention group and the control group (*P* > 0.05).

**Table 1:** Demographic characteristics of the intervention group and the control group.

**Variable**	**Intervention**	**Control**	***X*^**2**^/t (*P*)**
	**(*n* = 116)**	**(*n* = 95)**	
Child age, x ± s	7.93 ± 1.38	7.21 ± 1.22	0.645 (0.516)
Gender, *n* (%)			0.002 (0.812)
Boy	98 (84.5%)	81 (85.3%)	
Girl	18 (15.5%)	14 (14.7%)	
Grade, *n* (%)			2.321 (0.123)
<3 grade	40 (34.5%)	43 (45.3%)	
≥3 grade	75 (65.5%)	52 (55.7%)	
Primary caregiver, *n* (%)			0.798 (0.871)
Father	18 (15.5%)	16 (16.8%)	
Mother	78 (67.2%)	65 (68.4%)	
Grandparents	18 (15.5%)	11 (11.6%)	
Other	2 (1.7%)	3 (3.2%)	
Father's age, x ± s	36.68 ± 5.03	36.50 ± 5.37	0.639 (0.522)
Mother's age, x ± s	35.15 ± 4.14	34.82 ± 4.19	1.277 (0.536)
Father's education, *n* (%)			1.425 (0.487)
~Junior high school	27 (23.3%)	26 (27.4%)	
High school~ College	65 (56.0%)	54 (56.8%)	
College~	24 (20.7%)	15 (15.8%)	
Mother's education, *n* (%)			5.223 (0.071)
~Junior high school	32 (27.6%)	31 (32.7%)	
High school~ College	54 (46.6%)	51 (53.6%)	
College~	30 (25.8%)	13 (13.7%)	
Family structure, *n* (%)			1.727 (0.399)
Stem family	53 (45.7%)	39 (41.1%)	
Core family	56 (48.3%)	48 (50.5%)	
Single parent family	2 (1.7%)	5 (5.3%)	
Intergenerational family	5 (4.3%)	3 (3.1%)	
Family income/Yuan, *n* (%)			3.194 (0.526)
~5,000	8 (6.9%)	12 (12.7%)	
5,000–10,000	43 (37.1%)	34 (35.8%)	
10,000–15,000	27 (23.3%)	26 (27.3%)	
15,000–20,000	22 (19.0%)	14 (14.7%)	
20,000~	16 (13.7%)	9 (9.5%)	
**Duration of illness**			
~1 year	38 (32.8%)	29 (30.5%)	2.856 (0.275)
~2 years	26 (22.4%)	23 (24.2%)	
~3 years	25 (21.6%)	19 (20.00%)	
3 years~	27 (23.2%)	24 (25.3%)	
**Name of medication**			
Methylphenidate	75 (64.7%)	63 (66.3%)	1.387 (0.499)
Atomoxetine	41 (35.3%)	32 (33.7%)	

### Beliefs and Attitudes Toward Treatment Before and After Training

Among parents, only 29.2% believed that ADHD was a neurological disorder before training, while after training, 60.3% believed that. In addition, only 20.2% believed that medication was the first-line treatment for ADHD before training, while after training, 60.3% believed that. Among teachers, only 45.4% believed that ADHD was a neurological disorder before treatment, while after training, 75.1% believed that. Before training, only 32.6% believed that medication was the first line-treatment for ADHD, while after training, 67.3% believed that. The differences were statistically significant (Table 2). Beliefs and attitudes were also compared in control groups between baseline and 6-month follow-up, and there is no statistical significance.

**Table 2:** Beliefs and attitudes toward treatment before and after training (intervention group).

			**Before**	**After**	***_***X***_*^**2**^**	***P***
			**(%)**	**(%)**		
		Is a disorder	29.2	60.3	5.728	0.007
	Belief	Not a disorder	50.1	21.2	8.086	0.005
		Not sure	20.7	19.5	1.345	0.123
Parent		Medication	20.2	59.5	11.841	0.003
	Attitude (first line)	Behavior	41.3	30.4	3.336	0.086
		Psychotherapy	28.7	11.9	5.345	0.021
		Education	9.8	6.2	4.328	0.038
	Belief	Is a disorder	45.4	75.1	4.656	0.028
		Not a disorder	30.2	13.5	5.563	0.019
		Not sure	14.4	11.3	2.898	0.179
Teachers	Attitude (first line)	Medication	32.6	67.3	6.826	0.006
		Behavior	51.2	35.6	4.732	0.027
		Psychotherapy	10.2	4.8	5.437	0.020
		Education	6.0	5.3	3.678	0.102

### Medication Adherence at 6-Month Follow-Up in the Two Groups

**Figure 2:**
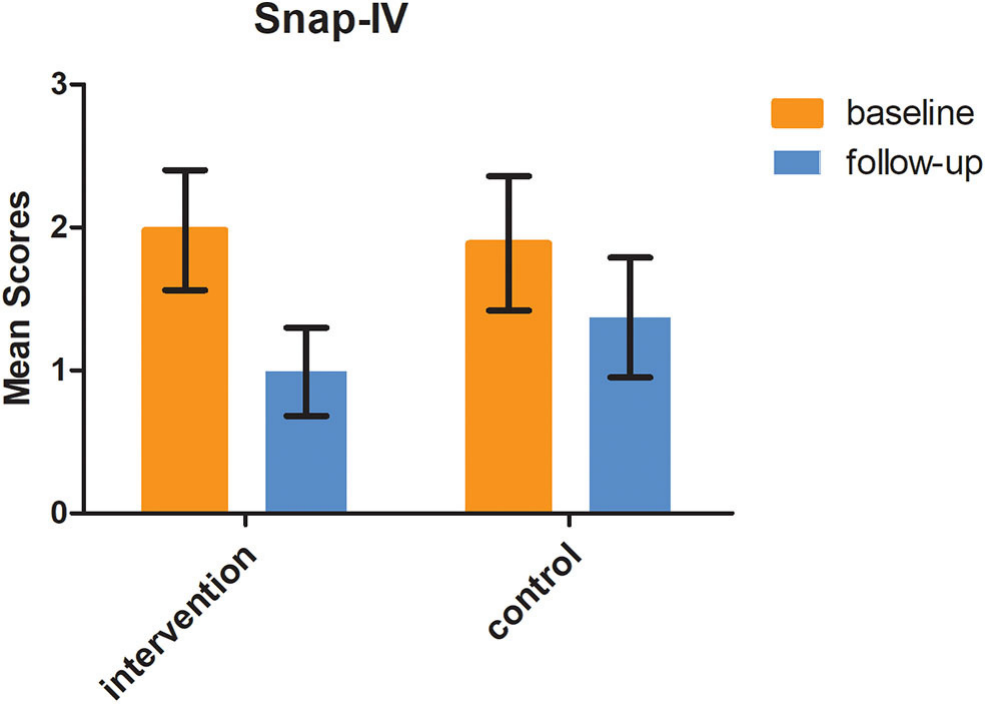
SNAP-IV scores at baseline and 6 months follow-up in the two groups (*F* = 2.67, *P* = 0.009).

A statistically significant difference was found in adherence measured by MARS. The MARS score for the intervention group was 22.8 (*SD* = 0.75), while it was 16.5 (*SD* = 1.63) for the control group (*t* = 5.217, *P* < 0.01), as shown in Figure 2. The difference was statistically significant.

**Figure 3:**
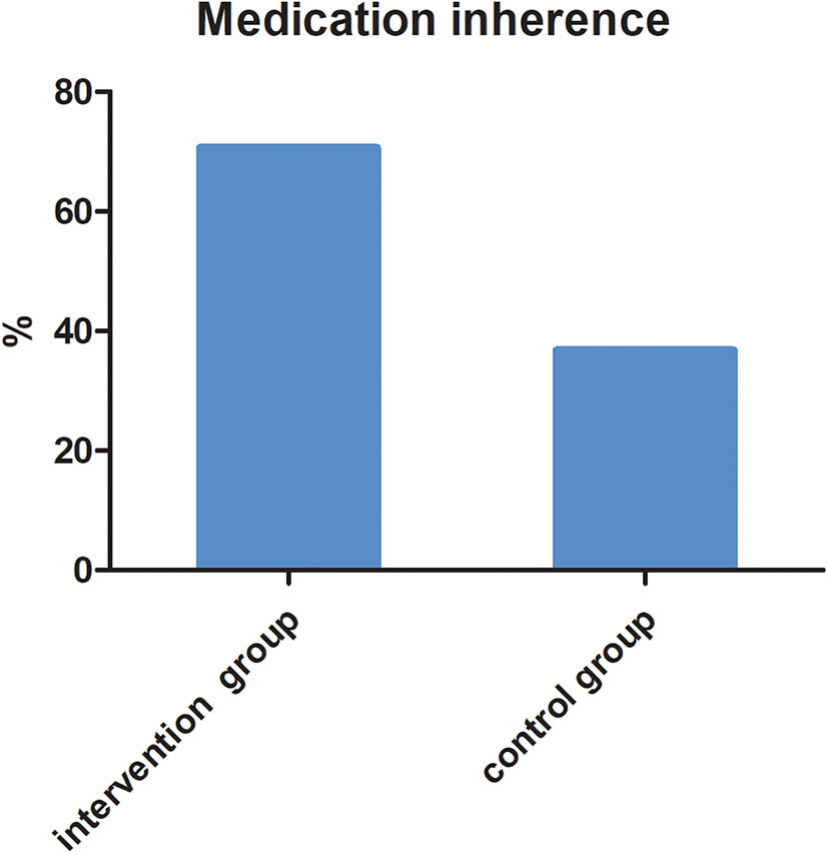
The proportion of children taking medication continuously for 6 months in two groups (*X*^2^ = 15.246, *P* = 0.001).

Taking medication continuously for 6 months based on parents' reports and medical records was considered good medication adherence. In the intervention group, there were 82 children (70.69%) continuously taking medication for 6 months, while in the control group, there were only 35 children (36.84%) (Figure 3). The difference was statistically significant (*X*^2^ = 15.246, *P* < 0.001).

Multiple regression analysis was used to determine the factors influencing medication adherence. They were parent beliefs, teacher beliefs, socioeconomic status, adverse effects, insurance coverage, gender, and trust of the medical system (Table 3).

**Table 3:** Factors influencing medication adherence for children with attention-deficit/hyperactivity disorder (ADHD).

**Variable**	***R***	**SE**	***Wald*x^**2**^**	***P***	**OR**	**95%CI**	
						**Upper**	**Lower**
Parents' belief	−3.634	1.384	6.895	0.002	0.026	0.002	0.398
Teachers' belief	−2.574	0.855	9.067	0.003	0.076	0.014	0.407
Socioeconomic status	−2.586	0.868	7.062	0.003	0.072	0.009	0.207
Adverse effect	−1.341	0.634	4.467	0.035	0.262	0.075	0.907
Insurance coverage	1.386	0.684	4.108	0.043	4.000	1.047	15.284
Gender	−1.934	1.084	5.891	0.005	0.026	0.004	0.296
Trust of medical system	−1.905	0.766	6.178	0.013	0.149	0.033	0.668

### Swanson Nolan and Pelham, Version IV Scale Scores at Baseline and 6-Month Follow-Up in the Two Groups

**Figure 4:**
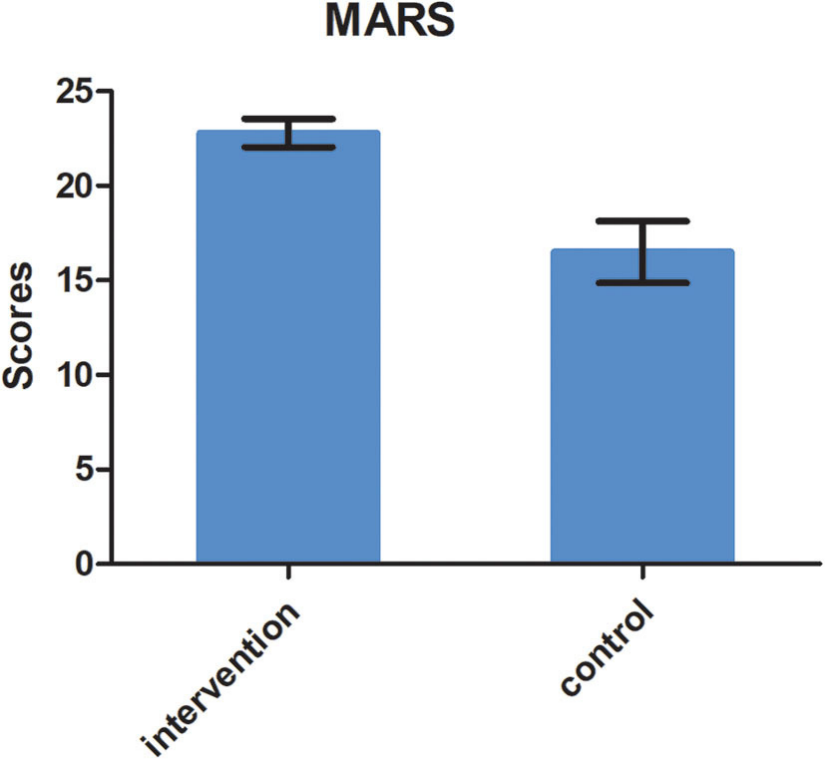
Medication adherence at 6 months follow-up in the two groups measured by MARS (*t* = 5.217, *P* = 0.01).

In the intervention group, the SNAP-IV score was 1.98 ± 0.42 at baseline, while it was 0.99 ± 0.31 at 6-month follow-up. In the control group, the SNAP-IV score was 1.89 ± 0.47 at baseline and 1.37 ± 0.42 at 6-month follow-up. The difference of SNAP-IV score changes between the two groups was statistically significant (*F* = 2.67, *P* = 0.009), as shown in Figure 4.

## Discussion

In our study, there were many more parents and teachers who believed that ADHD was a neurobiological disorder, and medication was first-line treatment after training. There are about 20 million children with ADHD in China, but less than one third of them receive medication. Among those children who use medication, only 10% take it continuously and for an extended period. That means only 3% of the children with ADHD take medicine continuously in China. Medication treatment is often intermittent or short term. Thus, in China many children with ADHD do not receive proper treatment (25). Parent and teacher training is necessary and urgent because the most significant determinant of medication initiation is parent belief about ADHD and attitudes toward treatment (26). For instance, some parents who believe that the child's difficulties are a medical disorder that requires a biological intervention will accept using medication and encourage long-term use (27). However, in China, many parents would like to choose behavioral strategies and other non-medication strategies, such as counseling, exercise, or dietary changes (28). A lot of parents often weigh concerns about social disapproval and adverse effects and have complex feelings about starting medication (29). They will take a long time to make a decision and miss the best treatment window. Good treatment effects also need the cooperation of teachers. Doctors need to refer to teachers' evaluation reports both in the evaluation and treatment periods. Teachers should know that the difficulties are a neurobiological disorder and should have the correct understanding of medication treatment. Therefore, the training should include teachers as well.

In our study, the MARS score in the intervention group was much higher than that in the control group, which suggested better adherence in the intervention group. The MARS contains only five items, so it is convenient and advantageous in clinical settings (30). As far as we know, the MARS has not been used to study medication adherence in ADHD in China. At 6-month follow-up, there were 82 children (70.69%) continuously taking medication for 6 months in the intervention group, and so we concluded that medication adherence was 70.69% in the intervention group. This finding is consistent with a previous study. Adherence has been reported to range between 20 and 81% in children and adolescents with ADHD (31). Medication adherence in ADHD varies considerably, depending on the method of measurement and sample characteristics.

Medication adherence consists of multidimensional factors related to the children, parent/family, health-care system, therapy, and socioeconomic circumstances (32). In our study, factors influencing medication adherence for children with ADHD were parent beliefs, teacher beliefs, socioeconomic status, adverse effects, insurance coverage, gender, and trust of the medical system. For children with ADHD, medication decisions are usually made by their parents, and so parent beliefs and attitudes toward ADHD are of critical importance. Charach and Fernandez further highlighted that parent beliefs about ADHD medication outweigh evidence of the real benefits and risks (33). It was found that parent beliefs and attitudes have more impact on medication use.

In our study, children with higher socioeconomic status had better adherence. This finding is consistent with other studies. In the United States, children from higher income groups are more likely to accept prescriptions and to use medication consistently than children from lower income groups (34).

Of course, adverse effects should be taken into consideration in medication adherence studies. Converging studies described concerns about potential short- and long-term adverse effects. It was reported that as many as 29% of children experienced non-serious adverse events with methylphenidate use (14). Prior notification of possible side effects along with preventive and therapeutic measures during parent and teacher training can help to increase adherence to medication. The better adherence in boys may be due to the more explicit and severe symptoms they experience.

Regarding the influence of the type of ADHD medication on adherence, our study found there was no difference between stimulants and non-stimulants. The evidence is limited and conflicting regarding the influence of the type of ADHD medication on adherence. Sixty-seven percent less adherence to immediate-release stimulant treatment compared with non-stimulant treatment has been found in 3- to 18-year-old children (35). Another study showed similar rates of medication adherence with stimulants vs. non-stimulants (36). At 6-month follow-up, the mean SNAP-IV score in the intervention group was much lower than that in the control group, suggesting that medication combined with parent–teacher behavior management is more effective in alleviating the core symptoms in children with ADHD (37). Behavioral interventions implemented by teachers at school and by parents at home are well-established for children with ADHD (38). Multimodal treatments are beneficial because they can help providers address impairments directly in multiple domains through parent–teacher collaboration. Recent findings emphasize the need to enhance training for parents and teachers of children with ADHD with strategies for increasing family and school involvement in their child's academic life (39).

The possible reasons for the increase in medication adherence when the parent and teacher trainings focused on education and behavior management components. There is evidence that parental acceptance is higher for combined and behavioral intervention approaches compared to medication alone, and parents like parent training components of intervention; this may have been part of why adherence was improved compared to the medication only group (40). In addition, there is research suggesting that implementation of behavioral intervention in combination with medication management may reduce medication dosages for treatment of ADHD, resulting in lower side effects and higher parental satisfaction with treatment (41). Therefore, children in the intervention group may have had lower medication dosages, resulting in fewer side effects and increased tolerability and acceptance by parents.

### Strengths and Limitations

The study provided a piloted, simplified, integrated training program for improving the knowledge, awareness, skill of management for ADHD children's parents and their teachers, which might help to facilitate the short-term clinical outcome and quality of life for children with ADHD.

There are several limitations in this study. The methodology for sampling and intervention in the study was single centered and district oriented, which limited the generalization of the conclusion. In addition, the evaluation tools for measuring the primary results were neither structured (questionnaire of beliefs and attitudes toward treatment) nor child-specific (MARS, five items), which weakened the interpretation of results. Risk factors identified to contribute to medication adherence were not validated because statistical results from multiple regression showed that OR value for insurance coverage only passed 1. Finally, information regarding the medication management of children in this study, both in the intervention and control groups, was not provided regarding changes in doses or medications utilized.

## Conclusion

Our study employed 4-week, session-based training for both parents and teachers and determined the medication adherence with questionnaire and score of rating scale baseline and endpoint of 6-month following up. The findings from our study indicate that comprehensive training programs improve the knowledge of ADHD and medication inherence for both children's parents and teachers, providing a promising approach for improving clinical efficacy for children with ADHD.

## Data Availability Statement

All datasets generated for this study are included in the article/Supplementary Material.

## Ethics Statement

The studies involving human participants were reviewed and approved by Ethics Committee of Shanghai Children's Hospital. Written informed consent to participate in this study was provided by the participants' legal guardian/next of kin.

## Author Contributions

XZ, GY, and YW contributed to the conception, data analysis, and writing. LJ contributed to the data analysis and editing of the manuscript. LS, XS, and YX contributed to the data analysis. All authors approved of the final submitted version of this manuscript.

## Conflict of Interest

The authors declare that the research was conducted in the absence of any commercial or financial relationships that could be construed as a potential conflict of interest.
