# Supplementary material for: Parent and Teacher Training Increases Medication Adherence for Primary School Children With Attention-Deficit/Hyperactivity Disorder
Source: Front Pediatr. 2020 Nov 9;8:486353. doi: 10.3389/fped.2020.486353 (PMC7680838; doi:10.3389/fped.2020.486353)
Supplement: Supplementary file 1 [file Data_Sheet_1.PDF]

Supplement 1

Questionnaire for knowledge about ADHD

Please answer these questions about your child according to specific situation. In this questionnaire, * represents multiple-choice questions and the rest are single-choice questions. Please attention not miss any question. Thank you very much.

1.What do you think ADHD is ?

(1) A disease (2) A bad habit (3) A character (4) I don't know .

2.* What do you think are the cause of ADHD ?

(1) Genetic factors (2) Biological factors (3) Family social environment (4) I don't know .

3.* What behaviors do you think are a sign of ADHD ?

(1) Inattention (2) Hyperactivity (3) Impulsivity, opposition and defiance (4) Making eyes and face.

4.* What do you think ADHD will affect ?

(1) Poor academic performance and carelessness (2) Social behavior problems (3)Teacher-student relationship (4) Parents-children relationship.

5.* What do you think are the treatment for ADHD ?

(1) Drug therapy (2) Behavioral-psychological therapy (3) Education and training (4)I don't know.

6.What do you think the treatment of ADHD needs ?

(1) Doctors' treatment (2) Teachers' education (3) Parents' urge (4) Cooperation of doctors, teachers and parents.

7.* What side effects do you think ADHD drugs have ?

(1) Poor appetite (2) Growth and development problems (3) Becoming stupid (4) Chronic diseases in adulthood.

8. * What are you most concerned about ADHD medication ?

(1) Addiction (2) Side effects (3)Only improve symptoms not root (4) I'm not worry because taking medication can help children.

9. What do you think is the prognosis of ADHD in children ?

(1) Children with ADHD will improve without treatment as growing up (2) Children with ADHD will get better if insisting on treatment (3) Children are ruined if diagnosed with ADHD (4) I don't know.

10..* What are your expectations for the treatment of ADHD ?

(1) Complete cure (2) Improve academic performance (3) Solve behavioral problems (4) Improve quality of life for parents and children.
